# 512 Mean Arterial Pressure, Morbidity, and Mortality Associated with Necrotizing Soft Tissue Infection

**DOI:** 10.1093/jbcr/irad045.109

**Published:** 2023-05-15

**Authors:** Holly Grossman, Brianna Marschke, Alan Pang, John Griswold, Deepak Bharadia

**Affiliations:** Texas Tech University Health Sciences Center, Lubbock, Texas; Texas Tech University Health Sciences Center, Lubbock, Texas; Texas Tech University Health Sciences Center, Lubbock, Texas; Texas Tech University Health Sciences Center, Lubbock, Texas; Texas Tech University Health Sciences Center, Lubbock, Texas; Texas Tech University Health Sciences Center, Lubbock, Texas; Texas Tech University Health Sciences Center, Lubbock, Texas; Texas Tech University Health Sciences Center, Lubbock, Texas; Texas Tech University Health Sciences Center, Lubbock, Texas; Texas Tech University Health Sciences Center, Lubbock, Texas; Texas Tech University Health Sciences Center, Lubbock, Texas; Texas Tech University Health Sciences Center, Lubbock, Texas; Texas Tech University Health Sciences Center, Lubbock, Texas; Texas Tech University Health Sciences Center, Lubbock, Texas; Texas Tech University Health Sciences Center, Lubbock, Texas; Texas Tech University Health Sciences Center, Lubbock, Texas; Texas Tech University Health Sciences Center, Lubbock, Texas; Texas Tech University Health Sciences Center, Lubbock, Texas; Texas Tech University Health Sciences Center, Lubbock, Texas; Texas Tech University Health Sciences Center, Lubbock, Texas; Texas Tech University Health Sciences Center, Lubbock, Texas; Texas Tech University Health Sciences Center, Lubbock, Texas; Texas Tech University Health Sciences Center, Lubbock, Texas; Texas Tech University Health Sciences Center, Lubbock, Texas; Texas Tech University Health Sciences Center, Lubbock, Texas

## Abstract

**Introduction:**

Adequate mean arterial pressure (MAP), or the average pressure in arteries during one cardiac cycle, plays an important role in ensuring adequate blood flow and perfusion in critically ill patients. MAP values of 65 mmHg or greater have been widely regarded as the ideal value for hospitalized patients in the past, especially those suffering from septic shock. Necrotizing soft tissue infections (NSTIs) present a unique challenge due to their rapid progression and high mortality, which creates the need for specific diagnostic and treatment guidelines that differ from those directed toward the care of sepsis secondary to other causes. Further research is still required to better understand the complex interactions between mean arterial pressure and morbidity and mortality among those with necrotizing soft tissue infections.

**Methods:**

Data was collected from a retrospective cohort study of 50 adult patients hospitalized with an NSTI from 2015-2021. MAP ranges in the first 48 hours of admission were sorted into four categories: under 60 mmHg, 60-69 mmHg, 70-79 mmHg, and above 79 mmHg. We investigated outcomes relating to increased morbidity, including need for repeat debridement, need for dialysis, or development of acute kidney injury. Statistical logistic regressions were fitted to model the outcome as a function of the available pool of predictors: demographic variables, co-morbidities, and hemodynamic variables, such as MAP and use of vasopressors.

**Results:**

In this small study, we found no significant association between time spent in different MAP groups or vasopressor volumes on the need for repeat debridement for NSTI patients or overall patient morbidity and mortality (p=0.1742). Patients who were significantly more likely to need a repeat debridement included those with previously diagnosed diabetes and hypertension (p=0.0485 and 0.0252, respectively).

**Conclusions:**

NSTI patients can tolerate mild fluctuations in MAP without these pressures significantly impacting their need for repeat debridement or other sources of morbidity. However, patients who are hypertensive at baseline or diabetic are significantly more likely to require a second surgery, necessitating a more extensive primary debridement.

**Applicability of Research to Practice:**

Patients hospitalized for NSTI can fluctuate at pressures below the established MAP goal of 65 mmHg without a significant need for pressors. Those with a history of hypertension or diabetes should be followed closely for possible spread of infection after primary surgery.

